# Metabolomics of Dry Versus Reanimated Antarctic Lichen-Dominated Endolithic Communities

**DOI:** 10.3390/life11020096

**Published:** 2021-01-27

**Authors:** Giuseppina Fanelli, Claudia Coleine, Federica Gevi, Silvano Onofri, Laura Selbmann, Anna Maria Timperio

**Affiliations:** 1 Department of Ecological and Biological Sciences, University of Tuscia, 01100 Viterbo, Italy; giuseppina.fanelli@unitus.it (G.F.); coleine@unitus.it (C.C.); gevi@unitus.it (F.G.); onofri@unitus.it (S.O.); 2 Italian National Antarctic Museum (MNA), Mycological Section, 16166 Genoa, Italy

**Keywords:** Antarctica, cryptoendolithic communities, untargeted metabolomics, adaptation, extremophiles, sun exposure

## Abstract

Cryptoendolithic communities are almost the sole life form in the ice-free areas of the Antarctic desert, encompassing among the most extreme-tolerant organisms known on Earth that still assure ecosystems functioning, regulating nutrient and biogeochemical cycles under conditions accounted as incompatible with active life. If high-throughput sequencing based studies are unravelling prokaryotic and eukaryotic diversity, they are not yet characterized in terms of stress adaptations and responses, despite their paramount ecological importance. In this study, we compared the responses of Antarctic endolithic communities, with special focus on fungi, both under dry conditions (i.e., when dormant), and after reanimation by wetting, light, and optimal temperature (15 °C). We found that several metabolites were differently expressed in reanimated opposite sun exposed communities, suggesting a critical role in their success. In particular, the saccharopine pathway was up-regulated in the north surface, while the spermine/spermidine pathway was significantly down-regulated in the shaded exposed communities. The carnitine-dependent pathway is up-regulated in south-exposed reanimated samples, indicating the preferential involvement of the B-oxidation for the functioning of TCA cycle. The role of these metabolites in the performance of the communities is discussed herein.

## 1. Introduction

Endolithic microbial communities are found inside rocks in the coldest or hottest drylands worldwide, from Atacama Desert in Chile to McMurdo Dry Valleys in Antarctica, where conditions of high solar radiation, drastic temperature fluctuations, water deficit, prolonged periods of desiccation, oligotrophy, and high salinity levels prevent the settlement of microbial epilithic patinas [1,2,3].

Scientific interest in endolithic microorganisms rose after their discovery in the McMurdo Dry Valleys, continental Antarctica, one of the harshest environments on Earth and accounted as a Terrestrial Martian analogue. Cryptoendoliths, due to their low complexity and diversity compared to other microbial consortia such as soil and biological crusts communities, are in fact excellent models to explore biotic and abiotic drivers of diversity, explore microbial communities responses under stress conditions, giving clues into the limit of life on Earth, and also for exploring the possibility for life elsewhere in the Solar System (e.g., Mars). The landscape of the McMurdo Dry Valleys is mostly composed of ice-free exposed rocks and oligotrophic mineral soils [4,5]. They are classified as a hyper-arid polar desert and are among the most extreme, coldest, and driest places on Earth. The mean annual air temperature is −20 °C [6]; typically there are less than 10 cm (water equivalent) of precipitation per year, restricted to only a few snowfall events [7,8]. The ability of a rock substrate to retain water is essential for its habitability [9]. Ertekin and coworkers [10] found that, regardless of water availability, substrate architecture was the driving factor that constrained microbial community diversity and function in gypsum endoliths of Atacama Desert. More recently, Huang et al. [11] showed that the microorganisms can extract water of crystallization (i.e., structurally ordered) from the rock, inducing a phase transformation from gypsum (CaSO_4_·2H_2_O) to anhydrite (CaSO_4_).

Endolithic communities are the main, if not the sole, life-forms representing the main standing biomass in the McMurdo Dry Valleys, occupying approximately 4% of sandstone boulders [12], up to 30% of granite boulders [13], and 100% of sandstone cliffs [1]. The most abundant and well characterized endolithic communities are those dominated by lichens [1,14] that are also a reservoir of endemic taxa [15,16,17,18]. These communities colonize porous rocks up to about 10 mm depth below the rock surface and microbial growth leads to differently colored bands, where each microbial component has different physiological adaptation and ecological abilities [1]. Light may penetrate few millimeters deep thanks to the translucence of quartzitic sandstone and photosynthetic algae, and cyanobacteria support a diversity of heterotrophic organisms [9,19,20,21]; these microorganisms all together are the main contributors to environmental/biogeochemical processes metabolizing C, N, and other macronutrients and mediate inputs and outputs of gases and nutrients and water uptake [12,22].

Among endolithic microorganisms, fungi are pivotal in these microbial consortia and are organized as follows. Lichen-forming fungi (class Lecanoromycetes, Ascomycota) are the most abundant [1,23], and the photobiotic counterpart is mostly represented by members of the green alga *Trebouxia* spp. (Chlorococcales) [24] that are considered the main source responsible for carbon fixation and sustain the entire community as primary producers. Black fungi or meristematic fungi (also known as rock-inhabiting fungi, RIF), in the classes Dothideomycetes, Eurotiomycetes, and Arthoniomycetes (Ascomycota), are consumers of nutrients from the community and play a primary role in protecting the whole community, forming a black “sunscreen” barrier just above the photobiont stratification [25]. Yeasts (orders Filobasidiales, Tremellales, and Cystobasidiales in Basidiomycota and Taphrinales in Ascomycota) are crucial in sustaining the community, as they produced various carotenoid pigments or mycosporines, playing a primary role in the response to oxidative stress (e.g., induced by UV radiation) [26]. Yeasts are also considered secondary consumers in the endolithic communities, utilizing traces of available substrates released by lysis of other community microbiota.

Recent studies are elucidating the biodiversity, structure, and composition of Antarctic cryptoendolithic communities [27,28,29,30,31,32,33,34], including their spatial organization [35] and the influence of environmental factors (i.e., altitude and sun exposure) on shaping biodiversity and community composition of functional groups of fungi [36]. However, despite their paramount ecological importance, Antarctic endolithic communities are not well characterized in terms of their genomic repertoire and stress adaptations. Only recently, Coleine et al. [37] generated the first metagenomes from rocks collected in Continental Antarctica over a distance of about 350 km along an altitudinal transect from 834 up to 3100 m a.s.l.; a total of 497 draft bacterial genomes were assembled and then clustered into 269 novel candidate species, functionally distinct from known related taxa species [17]. Friedmann and Ocampo-Friedmann [38] hypothesized that these microorganisms “hibernate” most of the time and activate the metabolism only when permissive climate conditions are reached in the summer season (e.g., when temperature rises, snow melts, and humidity increases), which anyway does not exceed 1000 h per year [39].

Microbial community metabolomics is recently providing a new route to establish stress-response and survival strategies in environmental samples [40,41]. A recent liquid chromatography (LC)—mass spectrometry (MS) based untargeted metabolomics—study compared the responses of two endolithic communities collected in the same locality in the McMurdo Dry Valleys, but subjected to very different environmental pressure due to south and north exposure of the rock surface [42]. The authors found specific responses and distinguished the two differently exposed communities, individuating mechanisms enabling the metabolic machinery to remain active under conditions that are lethal for the most organisms; in particular, expressed metabolites related to protection to solar irradiation of photosystems (allantoin) in the north exposed surface, while protectants to multiple stresses (melanin) in the south exposed surface.

In the present work, we evaluated the responses of differently stressed communities after reanimation by wetting, light, and temperature above freezing point, providing critical insights on metabolic processes under the extreme aridity and oligotrophy.

## 2. Materials and Methods

### 2.1. Sampling and Reanimation of Cryptoendolithic Communities

**Figure 1:**
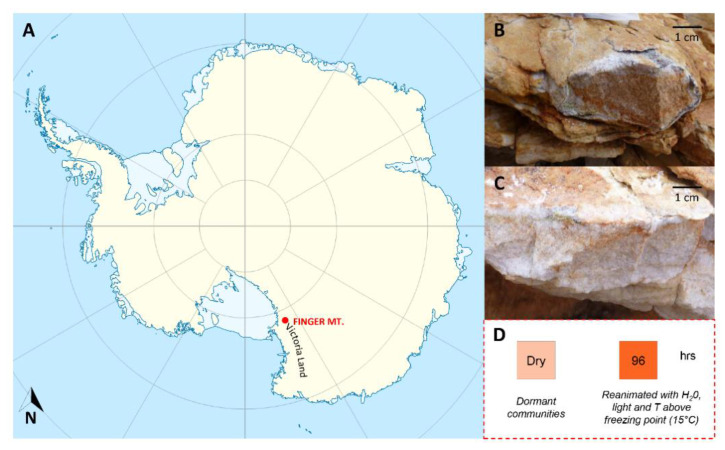
(**A**) Map of Antarctica. Red symbol indicates the study area, Finger Mt. (McMurdo Dry Valleys, Southern Victoria Land, Continental Antarctica); (**B**) Northern and (**C**) southern exposed rock surfaces; (**D**) outline of reactivation experiment. Credit by Italian National Antarctic Research Program (PNRA).

Three sandstone rocks colonized by cryptoendolithic communities were collected at Finger Mt. (1720 m a.s.l., McMurdo Dry Valleys, Southern Victoria Land, Continental Antarctica, Figure 1A) both from north (77°45′0.93” S 160°44’45.2” E) and south (77°45′10” S 160°44’44.39.7” E) exposed surfaces by L. Selbmann during the XXXI Italian Antarctic Expedition (Dec. 2015–Jan. 2016) (Figure 1B,C). Collecting dormant, differently sun-exposed communities allows us to fix the pattern of different stress responses in their natural habitat. The presence of endolithic colonization was assessed by direct observation in situ using magnification lenses. Rocks were excised using a geological hammer, placed in sterile bags, and shipped at −20 °C to the University of Tuscia (Italy), preserved at −20 °C in the Mycological Section of the Italian Antarctic National Museum (MNA), until downstream analysis.

To compare both response communities under dry conditions (i.e., when the community is dormant) and after metabolic machinery reactivation, samples have been exposed to wetting (3 mL sterile distilled water on round 5 cm^3^ of rock) and incubated for 96 hrs at 15 °C in an incubator (Panasonic MIR-254) equipped with a white light lamp (Figure 1D). After 96 hrs, rock samples were submerged in cold (−20 °C) HPLC grade methanol to quench metabolic activity [43], followed by rapid liquid nitrogen freezing and storage until metabolites extraction.

### 2.2. Metabolites Extraction

Protocols for a successful extraction of metabolites have been already optimized in a preliminary work we performed based on untargeted metabolomics, which aimed to explore the stress-response on Antarctic cryptoendolithic communities [42]. Briefly, 1 g of each crushed rock was added to 3000 μL of a chloroform/methanol/water (1:3:1 ratio) solvent mixture stored at −20 °C. Subsequently, samples were vortexed for 5 min and left on ice for 2 h for completed protein precipitation. The solutions were then centrifuged for 15 min at 15,000× *g*. and were dried to obtain visible pellets. Finally, the dried samples were re-suspended in 0.1 mL of water, 5% formic acid, and transferred to glass autosampler vials for LC/MS analysis.

Analysis has been performed in triplicate for each sample.

### 2.3. Ultra High-Performance Liquid Chromatography

Twenty μL of extracted supernatant samples was injected into an ultra-high-performance liquid chromatography (UHPLC) system (Ultimate 3000, Thermo) and run on a positive mode: samples were loaded on to a Reprosil C18 column (2.0 mm× 150 mm, 2.5 μm—Dr Maisch, Germany) for metabolite separation. Chromatographic separations were made at a column temperature of 30 °C and a flow rate of 0.2 mL/min. For positive ion mode (+) MS analyses, a 0–100% linear gradient of solvent A (ddH2O, 0.1% formic acid) to B (acetonitrile, 0.1%formic acid) was employed over 20 min, returning to 100% A in 2 min and holding solvent A for a 1-min post time hold. Acetonitrile, formic acid, and HPLC-grade water and standards (≥98% chemical purity) were purchased from Sigma Aldrich. The UHPLC system was coupled online with a Q Exactive mass spectrometer (Thermo) scanning in full MS mode (2 μscans) at resolution of 70,000 in the 67 to 1000 *m*/*z* range, a target of 1106 ions and a maximum ion injection time (IT) of 35ms with 3.8 kV spray voltage, 40 sheath gas, and 25 auxiliary gas. The system was operated in positive ion mode. Calibration was performed before each analysis against positive or negative ion mode calibration mixes (Pierce, Thermo Fisher, Rockford, IL) to ensure error of the intact mass within the sub ppm range.

### 2.4. Data Elaboration and Statistical Analysis

Raw files of metabolomics data from technical and biological replicates of south and north exposed samples both dry and reanimated samples were analyzed using XCMS Online, a freely accessible metabolite database called METLIN [44] (http://metlin.scripps.edu), which incorporates tandem mass spectral data from model compounds. Raw data sets were uploaded to XCMS Online, and a single group job was created to analyze the difference between the communities under dry conditions and after metabolic machinery reactivation in northern sun-exposed rocks. The raw data files are then processed for peak detection, retention-time correction, chromatogram alignment, metabolite feature metadata, and statistical evaluation using the predefined workflow settings for Orbitrap, and metabolite identification is facilitated through METLIN standard database matching and KEGG pathway database. XCMS extracted metabolomic features with statistically significant expression changes among the two groups to produce a list of raw differentially expressed features based on *p*-values (*p* ≤ 0.05, ≥ 1.5-fold change) and then perform pathway analyses directly from their raw metabolomic data. The output can be visualized through Pathway Cloud Plot. We then analyzed in detail only pathways with metabolites with *p* value ≤ 0.001.

## 3. Results

### 3.1. Different Community Response North Dry Samples vs. North Reanimated Samples

**Figure 2:**
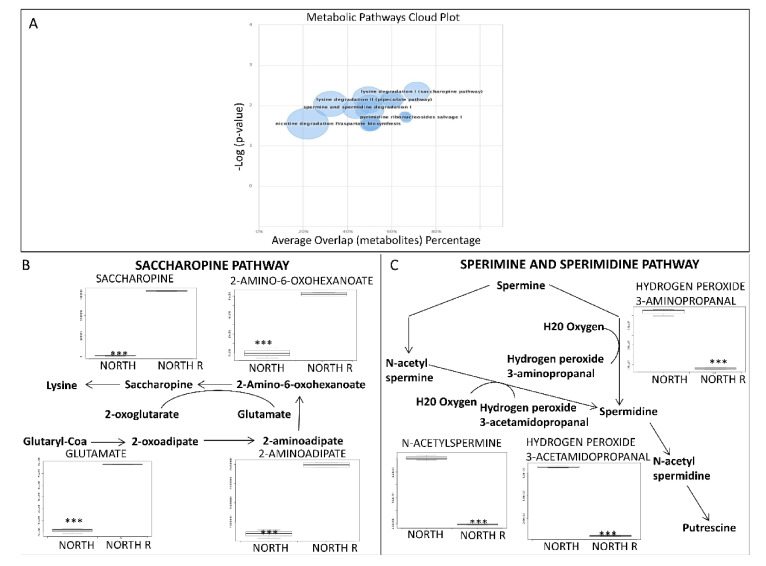
(**A**) The cloud plot shows perturbations in metabolism caused by the reactivation of northern exposed rock samples. Each pathway is displayed as a circle, with the *x* axis representing the percentage of metabolite overlap within that pathway and the *y* axis representing increased pathway significance calculated from the pathway analysis. The radius of each circle is proportional to the total number of metabolites in the pathway. Pathways with a higher percent overlap of metabolites and statistical significance will appear in the upper right corner (saccharopine pathway; spermine and spermidine degradation I; lysine-ketoglutarate reductase; pipecolate pathway). (**B**) Intermediates of the saccharopine pathway were up regulated after metabolic machinery reactivation. (**C**) Metabolomic profile of spermine and spermidine pathway. *p*-values less than 0.05 were considered significant; *p*-values marked *** *p* < 0.001.

XCMS software identified metabolites which were statistically significantly up- or down-regulated in reactivated northern sun-exposed rocks. Statistically different metabolic pathways in the two groups were identified and represented in a cloud plot (Figure 2A). The plot showed dysregulated pathways (blue circles) with increasing statistical significance on the *y* axis, metabolite overlapping on the *x* axis, and total number of metabolites in the pathway represented by the circle’s wideness.

We found that saccharopine pathway was up-regulated in reactivated samples; here, the alfa-aminoadipate is converted into 2-amino-6-oxohexanoate, which is then condensed with glutamate into saccharopine, which is finally hydrolyzed to form lysine (Figure 2B).

Conversely, the spermine and spermidine pathway is down-regulated in reactivated samples (Figure 2C). In fungi, the putrescine, precursor to spermidine and spermine, is synthesized from amino acid ornithine (ADC) via ornithine decarboxylase (ODC). The first step is the acetylation of the aminopropyl group of polyamines, a reaction catalyzed by spermine or spermidine N1-acetyltrasferase to give either N-acetyl-spermidine or N-acetyl-spermine. These are degraded by a polyamine oxidase, with the formation of either putrescine or spermidine (Figure 2C). In our work, we showed that the polyamine pathway is turned off in the reactivated samples; a significant increase of hydroxy peroxide 2 aminopropanol in n-acetyl spermidine and hydroxy peroxide 3-acetamido-propanal was observed.

### 3.2. Different Community Response South Dry Samples vs. South Reanimated Samples

**Figure 3:**
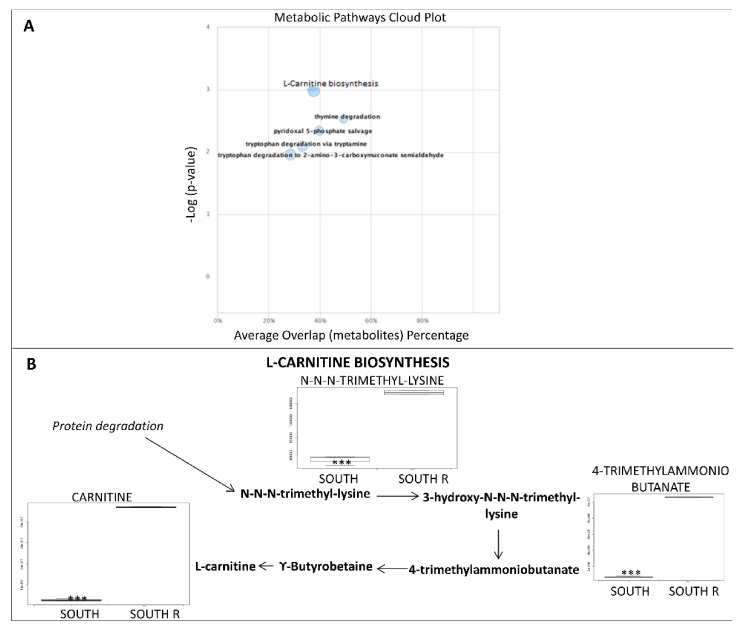
(**A**) The cloud plot shows perturbations in cellular metabolism caused by the reactivation of southern exposed rock samples. Each pathway is displayed as a circle, with the *x* axis representing the percentage of metabolite overlap within that pathway and the *y* axis representing increased pathway significance calculated from the pathway analysis. The radius of each circle is proportional to the total number of metabolites in the pathway. Pathways with a greater percent overlap of metabolites and statistical significance are found in the upper right corner. (**B**) Overview of L-carnitine biosynthesis metabolism. *p*-values < 0.05 are considered significant; *p*-values marked *** *p* < 0.001.

The most significant pathway up-regulated in reanimated south exposed samples is related to carnitine biosynthesis (Figure 3A,B). L-carnitine is synthesized from the essential amino acid lysine via a specific biosynthetic pathway; lysine is methylated to form ε-N-trimethyllysine in a reaction catalyzed by specific lysine methyltransferases that use S-adenosyl-methionine (derived from methionine) as a methyl donor. Then, ε-N-Trimethyllysine is released for carnitine synthesis by protein hydrolysis.

### 3.3. Different Community Response North Reanimated Samples vs. South Reanimated Samples

**Figure 4:**
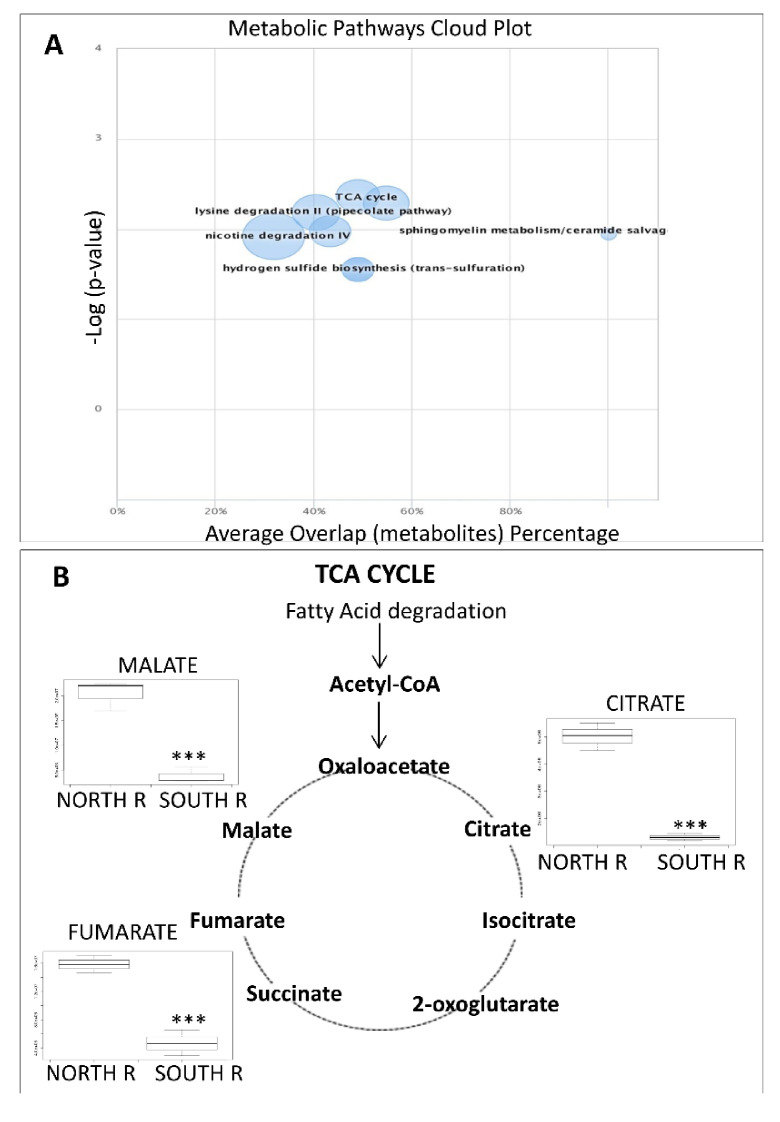
(**A**) The cloud plot shows perturbations in cellular metabolism between northern and southern exposed reactivated rocks. Each pathway is displayed as a circle, with the *x* axis representing the percentage of metabolite overlap within that pathway and the *y* axis representing increased pathway significance calculated from the pathway analysis. The radius of each circle is proportional to the total number of metabolites in the pathway. Tricarboxylic acid cycle (TCA) with a greater percent overlap of metabolites and statistical significance is found in the upper right corner. (**B**) Intermediates of TCA cycle measured in northern and southern exposed reactivated rocks. *p*-values < 0.05 are considered significant; *p*-values marked *** *p* < 0.001.

We also investigated differences/similarities in responses between northern and southern reanimated rock samples. Figure 4A shows that the most influenced is the tricarboxylic acids (TCA) pathway. In particular, we found that the southern reactivated samples prefer the TCA pathway to restore their function. An oxidation of the metabolic intermediates related to the TCA such as citrate, fumarate, and malate is shown in Figure 4B.

## 4. Discussion

Climate change is particularly pronounced and challenging in Polar and dryland ecosystems. Understanding the mechanisms underlying microbial resistance and resilience to harsh conditions is essential to predict the fate of these weak ecosystems in a warming and drying world. However, the adaptation mechanisms of microbial communities to natural perturbations remain relatively unexplored, particularly in extreme environments, limiting our possibility to effectively model and predict the responses of microbiomes to external stressors.

Cryptoendolithic microbial lichen-dominated communities are the predominant life-form in the McMurdo Dry Valleys, an ice-free area covering the 2% of the Antarctic continent characterized by harshest conditions of drought, oligotrophy, low temperature, and high UV irradiation.

The growth of endolithic colonization is strongly influenced by high-frequency temperature oscillations around 0 °C, which generate freeze–thaw cycles that are believed to contribute to the abiotic nature of rock surfaces [45]. Although temperature extremes are commonly thought to be the major limiting factor for life in Antarctic desert, moisture may play a more important role in determining the survival of these microbial consortia.

Despite the harshest climate conditions of McMurdo Dry Valleys, our recent study clearly indicated that active metabolism can still occur, revealing that microorganisms in this unique ecological niche developed several adaptation strategies in terms of key metabolites produced, according to increasing environmental pressure [42]. This study supported the hypothesis that they can maximize the metabolic activity and reproduction, even taking advantage of the moisture retained inside the rock [46]. Otherwise, for most of the time they remain dormant [39]. Even in the driest parts of the Atacama Desert, Davila et al. [47] reported microbial communities metabolically active in specialized microhabitats such as the interior of salt nodules, forming a complete and self-sustainable community. Prolonged drought or optimal conditions can have a significant impact on the activity of endolithic microbial communities, and changes in moisture may significantly influence the functionality of microorganisms and the processes they control.

Therefore, the main aims of the present study were to (i) compare the effect of reanimation (i.e., when conditions are optimal for microbial activity) on metabolism and response of Antarctic endolithic communities with those under dry conditions (i.e., when community is dormant) and (ii) reveal which metabolic pathways, with a special focus on fungi as main component of these communities, play a critical role for reactivation.

We found that a quite diverse activity of microbial communities in these two conditions, resulting in a different magnitude of change to the overall functioning, can be discerned by comparing biochemical pathways in (i) North dry samples (NDS) vs. North reanimated samples (NRS), (ii) South dry samples (SDS) vs. South reanimated samples (SRS), and (iii) NRS vs. SRS.

One of the main differences found concerned the NDS vs. NRS comparison, where the “saccharopine” and “spermine and spermidine” pathways resulted in strongly up-regulated in reactivated samples. In fungi, lysine is synthesised through the “saccharopine pathway”; this pathway also occurs in higher plants and animals, but instead of functioning in lysine synthesis, it works in the catabolic reaction, leading to lysine degradation [48]. The up-regulation of this pathway in NRS confirms the activation of this metabolism to produce lysine. Indeed, several fungal alkaloids or peptides have lysine as a structural element or biosynthetic precursor. The a-aminoadipate (AAA) pathway for lysine biosynthesis is unique to fungi. The presence of the AAA pathway for lysine biosynthesis has been demonstrated in several fungi, including *Aspergillus fumigatus* and a few yeasts such as *Saccharomyces cerevisiae, Yarrowia lipolytica, Schizosaccharomyces pombe,* and *Rhodotorula glutinis.* Members of *Rhodotorula* genus were also retrieved from halite nodules from samples collected in the Atacama desert; in this work, Aspergillaceae, Sporidiobolaceae, and Sordariaceae families were the most highly represented in these shotgun metagenome sequences [49].

Additionally, enzymes involved in the fungal AAA pathway are unique to lysine synthesis; in addition, several saccharopine pathway intermediates are incorporated into secondary metabolites [50] that are directly involved in the growth, development, and interactions with other components of the community or reproduction that may be activated when communities are reanimated.

On the contrary, the performance of microbial endolithic communities subjected to abiotic stress (dormant state) requires a concerted orchestration of the multiple cellular and metabolic remodeling processes. We found, indeed, that the lysine synthesis was strongly inhibited by water deficit, contrary to what we observed in NRS. Drought, indeed, induced substantial shifts in NRS, resulting in an up-regulation of the “spermine and spermidine” biosynthesis, showing the important role of metabolites involved in this pathway in protecting microbial activity in drought periods. Spermine and spermidine are polyamines that are essential metabolites present in all living organisms, and they act in different ways in the cell functions in which they are involved, from growth to development and differentiation and protection of DNA from reactive oxygen species (ROS). During the last decades, fungi have contributed to the understanding of polyamine metabolism; a few years ago, the use of specific inhibitors and the isolation of mutants have allowed the manipulation of the pathway providing information on its regulation [51]. The polyamines, of which putrescine (a precursor of spermine) is one of the simplest, appear to be growth factors necessary for cell division. For instance, when putrescine biosynthesis was inhibited by DFMO (D, L-α-difluoromethylornithine), growth of the fungus *Colletotrichum truncatum* was inhibited [52]. The same result was obtained by the use of DFMO on other economically important plant pathogens such as *Rhizoctonia solani, Fusarium oxysporum, and Cochliobolus carbonum* [53]. Additionally, several studies [54,55,56] reported that polyamines have multiple protective roles in cell survival under extreme conditions such as temperature, drought, oxidative, and osmotic stresses. The up-regulation of this pathway in NDS may be related to the role of polyamines in stress-response to the natural conditions.

When comparing dry and reanimated samples collected in the southern exposed surfaces, we reported an alteration of “L-carnitine biosynthesis pathway” in SRS. Carnitine biosynthesis led to the endogenous production of L-carnitine, a molecule that is essential for energy metabolism that is necessary when rocks are reanimated and their metabolic machinery is reactivated; conversely, in a dormant state, the microorganisms decrease their energetic costs. The carnitine biosynthesis pathway is highly conserved among many eukaryotes and some prokaryotes, although it acts in diverse physiological processes. In this work, we reconstructed the L-carnitine pathway from the precursor *N*^ε^-trimethyl-lysine biosynthetic pathway that has been fully characterized in fungi including *Neurospora crassa*. Genetic studies in the yeast *Candida albicans* led to the identification of the aldolase that catalyses the second step of the carnitine biosynthesis pathway, suggesting that fungi utilize this pathway to produce carnitine for the β-oxidation [57]. Indeed, Fatty acid β-oxidation is completely peroxisomal in fungi, and therefore acetyl units are the only products that need to be transported from peroxisomes to mitochondria. It was reported that black cryptoendolithic fungi may store high levels of fats as a reservoir of energy, and the up regulation of this pathway may be, therefore, involved in energy production in reanimated samples. Lipids in fungi are synthesized at the endoplasmic reticulum and stored in lipid droplets [58]. In addition to acting as energy storage, they provide precursor molecules for fatty acid and phospholipid synthesis, lipoproteins, and lipid-signaling molecules [59,60]. They also have a role beyond lipid metabolism, impacting protein degradation, apoptosis, virulence, immunity, and, as here may be the case, even stress responses [61].

Finally, we compared the reanimation processes between northern and southern exposed communities. Recently, it has been reported that abundance and composition of metabolites presented a clear pattern of correlation across opposite exposed surfaces of the same mountain, revealing significant changes in metabolic profiles following sunlight deprivation [42]. Overall, the notable variability observed across the two exposures suggested that this abiotic parameter as a driving factor in shaping both the fungal community composition and structure and survival strategies in the Antarctic cryptoendolithic communities [36]. Warming of cryptoendolithic habitats occurs primarily through solar heating [7,39]. Indeed, it has been widely observed that northern sun exposed rock surfaces are warmer than air temperatures during periods of insolation due to the heat capacity and transparency of the rock as well as trapping of heat within the subsurface, which can be up to 20 °C higher than air temperatures [45]; in some sites of the McMurdo Dry Valleys, climatic conditions are so extreme in climate that orientation of the rock surface can determine whether colonization is possible or not. This enhanced heating of the endolithic niche increases the time during which water is available and microorganisms can be metabolically active.

In the present study, we found that the endolithic communities exposed directly to the sun (northern surface) use canonical pathways for energy production compared to the shady samples, suggesting that the latter may preferentially use alternative mechanisms, more appropriate to keep metabolism active under more unfavorable conditions.

By comparing responses of *North* vs. *South reanimated samples,* the TCA cycle appears significantly perturbed. As discussed above, SRS use the carnitine-dependent pathway, where the carnitine acetyltransferases exchange the CoA group of acetyl-CoA for carnitine, thereby forming acetyl-carnitine, which can be transported in peroxisome for the B-oxidation. Then, the products of the peroxisomal b-oxidation (shortened fatty acids and acetyl units) were transported to the mitochondrial TCA for ATP generation that are essential for growth and survival [62] and that results up-regulated in these samples as a consequence. Black cryptoendolithic fungi, particularly abundant in the south exposed endolithic communities [36], may store high levels of fats as a reservoir of energy justifying the observed up regulation of this pathway in SRS.

Based on these results, we may hypothesize that the two opposite sun-exposed communities activate different metabolic pathways to restore their vitality and that the south-exposed ones, being more stressed, prefer peroxisomal oxidation (through biosynthesis of carnitine) and TCA cycle as the main pathways for producing energy.

## 5. Conclusions

Cryptoendolithic microbial ecosystems have unique properties as a model system for studies of microbial ecology in both hot and cold deserts. In this study, we provided critical insights on how Antarctic endolithic communities respond to stresses, maintaining biological activities under harshest conditions that are typically incompatible with active life. We also identified metabolites that might be expressed in response to optimal conditions (after reanimation), characterizing the response dynamics of these communities to changing environmental conditions. Our results show that endolithic microorganisms, in particular fungi, exist in dormant states but rapidly reactivate the saccharopine and polyamines biosynthesis pathways that are tightly correlated with the growth, development, or reproduction of the organisms and energy metabolism and may play an important role when the communities face environmental changes in its living habitat.

Although further research on a larger number of samples is necessary to attribute shifts in particular microbially driven endolithic processes to the observed changes in functional and genetic diversity under different conditions, these findings will be useful in modelling predicting important pathways that have evolved to support these life-forms on the extreme edge of Antarctic desert.

These results may be also applied to microbial endolithic ecosystems in drylands worldwide, in an era of climate change and rapid desertification.

## References

[1] Friedmann E.I. (2015). Endolithic microorganisms in the Antarctic cold desert. Science.

[2] Cary S.C., McDonald I.R., Barrett J.E., Cowan D.A. (2010). On the rocks: The microbiology of Antarctic Dry Valley soils. Nat. Rev. Microbiol..

[3] Pointing S.B., Belnap J. (2012). Microbial colonization and controls in dryland systems. Nat. Rev. Microbiol..

[4] Ugolini F.C., Bockheim J.G. (2008). Antarctic soils and soil formation in a changing environment: A review. Geoderma.

[5] Lee J.R., Raymond B., Bracegirdle T.J., Chadès I., Fuller R.A., Shaw J.D., Terauds A. (2017). Climate change drives expansion of Antarctic ice-free habitat. Nature.

[6] Doran P.T., McKay C.P., Clow G.D., Dana G.L., Fountain A.G., Nylen T., Lyons W.B. (2002). Valley floor climate observations from the McMurdo Dry Valleys, Antarctica, 1986–2000. J. Geophys. Res. Atmosph..

[7] Omelon C.R., Pollard W.H., Ferris F.G. (2006). Environmental controls on microbial colonization of high Arctic cryptoendolithic habitats. Polar Biol..

[8] Fountain A.G., Nylen T.H., Monaghan A., Basagic H.J., Bromwich D. (2010). Snow in the McMurdo dry valleys, Antarctica. Int. J. Clim..

[9] Wierzchos J., de los Ríos A., Ascaso C. (2012). Microorganisms in desert rocks: The edge of life on Earth. Int. Microbiol..

[10] Ertekin E., Meslier V., Browning A., Treadgold J., DiRuggiero J. (2020). Rock structure drives the taxonomic and functional diversity of endolithic microbial communities in extreme environments. Environ. Microbiol..

[11] Huang W., Ertekin E., Wang T., Cruz L., Dailey M., DiRuggiero J., Kisailus D. (2020). Mechanism of water extraction from gypsum rock by desert colonizing microorganisms. Proc. Natl. Acad. Sci. USA.

[12] Pointing S.B., Chan Y., Lacap D.C., Lau M.C., Jurgens J.A. (2009). Highly specialized microbial diversity in hyper arid polar desert. Proc. Natl. Acad. Sci. USA.

[13] Yung C.C.M., Chan Y., Lacap D.C., Pérez-Ortega S., de los Rios-Murillo A., Lee C.K., Cary S.C., Poiting S.B. (2014). Characterization of chasmoendolithic community in Miers Valley, McMurdo Dry Valleys, Antarctica. Microb. Ecol..

[14] Coleine C., Stajich J.E., de Los Ríos A., Selbmann L. (2020). Beyond the extremes: Rocks as ultimate refuge for fungi in drylands. Mycologia.

[15] Selbmann L., de Hoog G.S., Mazzaglia A., Friedmann E.I., Onofri S. (2005). Fungi at the edge of life: Cryptoendolithic black fungi from Antarctic deserts. Stud. Mycol..

[16] Selbmann L., de Hoog G.S., Zucconi L., Isola D., Ruisi S., Gerrits van den Ende A.H.G., Ruibal C., De Leo F., Urzì C., Onofri S. (2008). Drought meets acid: Three new genera in a dothidealean clade of extremotolerant fungi. Stud. Mycol..

[17] Albanese D., Coleine C., Rota-Stabelli O., Onofri S., Tringe S., Stajich J.E., Selbmann L., Donati C. (2020). Antarctic cryptoendolithic bacterial lineages of pre-Cambrian origin as proxy for Mars colonization. bioRxiv.

[18] Coleine C., Pombubpa N., Zucconi L., Onofri S., Stajich J.E., Selbmann L. (2020). Endolithic fungal species markers for harshest conditions in the McMurdo Dry valleys, Antarctica. Life.

[19] Walker J.J., Pace N.R. (2007). Endolithic microbial ecosystems. Annu. Rev. Microbiol..

[20] Meslier V., Casero M.C., Dailey M., Wierzchos J., Ascaso C., Artieda O., McCullough P.R., DiRuggiero J. (2018). Fundamental drivers for endolithic microbial community assemblies in the hyperarid Atacama Desert. Environ. Microbiol..

[21] Meslier V., DiRuggiero J. (2019). Endolithic microbial communities as model systems for ecology and astrobiology. Model Ecosystems in Extreme Environments.

[22] Makhalanyane T.P., Valverde A., Velázquez D., Gunnigle E., Van Goethem M.W., Quesada A., Cowan D.A. (2015). Ecology and biogeochemistry of cyanobacteria in soils, permafrost, aquatic and cryptic polar habitats. Biodivers. Conserv..

[23] Selbmann L., Onofri S., Coleine C., Buzzini P., Canini F., Zucconi L. (2017). Effect of environmental parameters on biodiversity of the fungal component in the lithic Antarctic communities. Extremophiles.

[24] Perez-Ortega S., Ortiz-Álvarez R., Allan Green T.G., de los Ríos A. (2012). Lichen myco-and photobiont diversity and their relationships at the edge of life (McMurdo Dry Valleys, Antarctica). FEMS Microbiol. Ecol..

[25] Selbmann L., Grube M., Onofri S., Isola D., Zucconi L. (2013). Antarctic epilithic lichens as niches for black meristematic fungi. Biology.

[26] Vishniac H.S., Hempfling W.P. (1979). Evidence of an indigenous microbiota (yeast) in the dry valleys of Antarctica. Microbiology.

[27] de la Torre J.R., Goebel B.M., Friedmann E., Pace N.R. (2003). Microbial diversity of cryptoendolithic communities from the McMurdo Dry Valleys. Antarctica. Appl. Environ. Microbiol..

[28] Archer S.D., de los Ríos A., Lee K.C., Niederberger T.S., Cary S.C., Coyne K.J., Douglas S.C., Lacap-Bugler D.C., Pointing S.B. (2017). Endolithic microbial diversity in sandstone and granite from the McMurdo Dry Valleys, Antarctica. Polar Biol..

[29] Qu E.B., Omelon C.R., Oren A., Meslier V., Cowan D.A., Maggs-Kölling G., DiRuggiero J. (2020). Trophic selective pressures organize the composition of endolithic microbial communities from global deserts. Front. Microbiol..

[30] Coleine C., Stajich J.E., Zucconi L., Onofri S., Pombubpa N., Egidi E., Franks A., Buzzini P., Selbmann L. (2018). Antarctic cryptoendolithic fungal communities are highly adapted and dominated by Lecanoromycetes and Dothideomycetes. Front. Microbiol..

[31] Coleine C., Stajich J.E., Pombubpa N., Zucconi L., Onofri S., Canini F., Selbmann L. (2019). Altitude and fungal diversity influence the structure of Antarctic cryptoendolithic Bacteria communities. Environ. Microbiol. Rep..

[32] Coleine C., Masonjones S., Sterflinger K., Onofri S., Selbmann L., Stajich J.E. (2020). Peculiar genomic traits in the stress-adapted cryptoendolithic Antarctic fungus *Friedmanniomyces endolithicus*. Fungal. Biol..

[33] Coleine C., Pombubpa N., Zucconi L., Onofri S., Turchetti B., Buzzini P., Stajich J.E., Selbmann L. (2020). Uncovered Microbial Diversity in Antarctic Cryptoendolithic Communities Sampling three Representative Locations of the Victoria Land. Microorganisms.

[34] Coleine C., Stajich J.E., Zucconi L., Onofri S., Selbmann L. (2020). Sun exposure drives Antarctic cryptoendolithic community structure and composition. Polar Biol..

[35] De Los Ríos A., Wierzchos J., Ascaso C. (2014). The lithic microbial ecosystems of Antarctica’s McMurdo Dry Valleys. Antarct. Sci..

[36] Coleine C., Zucconi L., Onofri S., Pombubpa N., Stajich J.E., Selbmann L. (2018). Sun exposure shapes functional grouping of fungi in cryptoendolithic Antarctic communities. Life.

[37] Coleine C., Albanese D., Onofri S., Tringe S.G., Pennacchio C., Donati C., Stajich J.E., Selbmann L. (2020). Metagenomes in the borderline ecosystems of the Antarctic cryptoendolithic communities. Microbiol. Resour. Announc..

[38] Friedmann E.I., Ocampo R. (1976). Endolithic blue-green algae in dry valleys-primary producers in Antarctic desert ecosystem. Science.

[39] Friedmann E.I., McKay C.P., Nienow J.A. (1987). The cryptoendolithic microbial environment in the Ross Desert of Antarctica: Satellite-transmitted continuous nanoclimate data, 1984 to 1986. Polar Biol..

[40] Gargallo-Garriga A., Ayala-Roque M., Sardans J., Bartrons M., Granda V., Sigurdsson B.D., Leblans N.I.W., Oravec M., Urban O., Janssens U.A. (2017). Impact of soil warming on the plant metabolome of icelandic grasslands. Metabolites.

[41] Jeppe K.J., Kouremenos K.A., Townsend K.R., MacMahon D.F., Sharley D., Tull D.L., Hoffmann A.A., Pettigrove V., Long S.M. (2017). Metabolomic profiles of a midge (*Procladius villosimanus*, kieffer) are associated with sediment contamination in urban wetlands. Metabolites.

[42] Coleine C., Gevi F., Fanelli G., Onofri S., Timperio A.M., Selbmann L. (2020). Specific adaptations are selected in opposite sun exposed Antarctic cryptoendolithic communities as revealed by untargeted metabolomics. PLoS ONE.

[43] Sapcariu S.C., Kanashova T., Weindl D., Ghelfi J., Dittmar G., Hiller K. (2014). Simultaneous extraction of proteins and metabolites from cells in culture. Methods.

[44] Smith C.A., O’Maille G., Want E.J., Qin C., Trauger S.A., Brandon T.R., Custodio D.E., Abagyan R., Siuzdaket G. (2005). METLIN: A metabolite mass spectral database. Ther. Drug Monit..

[45] McKay C.P., Nienow J.A., Meyer M.A., Friedmann E.I., Bromwich D.H., Stearns C.R. (1993). Continuous nanoclimate data (1985–1988) from the Ross Desert (McMurdo Dry Valleys) cryptoendolithic microbial ecosystem. Antarctic Meteorology and Climatology: Studies Based on Automatic Weather Stations.

[46] Nienow J.A., Friedmann E.I., Friedmann E.I. (1993). Terrestrial lithophytic (rock) communities. Antarctic Microbiology.

[47] Davila A.F., Hawes I., Araya J.G., Gelsinger D.R., DiRuggiero J., Ascaso C., Osano A., Wierzchos J. (2015). In situ metabolism in halite endolithic microbial communities of the hyperarid Atacama Desert. Front. Microbiol..

[48] de Mello Serrano G.C., Kiyota E., Zanata N., Arruda P. (2012). Lysine degradation through the saccharopine pathway in bacteria: LKR and SDH in bacteria and its relationship to the plant and animal enzymes. FEBS Lett..

[49] Gómez-Silva B., Vilo-Muñoz C., Galetović A., Dong Q., Castelán-Sánchez H.G., Pérez-Llano Y., Sánchez-Carbente M.d.R., Dávila-Ramos S., Cortés-López N.G., Martínez-Ávila L. (2019). Metagenomics of Atacama Lithobiontic Extremophile Life Unveils Highlights on Fungal Communities, Biogeochemical Cycles and Carbohydrate-Active Enzymes. Microorganisms.

[50] Xu H., Andi B., Qian J., West A.H., Cook P.F. (2006). The α-aminoadipate pathway for lysine biosynthesis in fungi. Cell Biochem. Biophys..

[51] Valdés-Santiago L., Cervantes-Chávez J.A., León-Ramírez C.G., Ruiz-Herrera J. (2012). Polyamine metabolism in fungi with emphasis on phytopathogenic species. J. Amino Acids.

[52] Gamarnik A., Frydman R.B., Barreto D. (1994). Prevention of infection of soybean seeds by *Colletotrichum truncatum* by polyamine biosynthesis inhibitors. Phytopathology.

[53] Kumria R., Virdi J.S., Rajam M.V. (2000). Increasing the efficacy of difluoromethylornithine to inhibit the growth of three phytopathogenic fungi by membrane modifying agents. Curr. Sci..

[54] Alcázar R., Altabella T., Marco F., Bortolotti C., Reymond M., Koncz C., Carrasco P., Tiburcio A.F. (2010). Polyamines: Molecules with regulatory functions in plant abiotic stress tolerance. Planta.

[55] Gupta K., Dey A., Gupta B. (2013). Plant polyamines in abiotic stress responses. Acta Physiol. Plant.

[56] Valdés-Santiago L., Ruiz-Herrera J. (2014). Stress and polyamine metabolism in fungi. Front. Chem..

[57] Strijbis K., Van Roermund C.W., Hardy G.P., Van den Burg J., Bloem K., de Haan J., Van Vlies N., Ronald J.A., Wanders R.J.A., Vaz F.M. (2010). Identification and characterization of a complete carnitine biosynthesis pathway in *Candida albicans*. FASEB J..

[58] Coleman R.A., Lee D.P. (2004). Enzymes of triacylglycerol synthesis and their regulation. Prog. Lipid Res..

[59] Guo Y., Cordes K.R., Farese R.V., Walther T.C. (2009). Lipid droplets at a glance. J. Cell Sci..

[60] Murphy D.J. (2012). The dynamic roles of intracellular lipid droplets: From archaea to mammals. Protoplasma.

[61] Keyhani N.O. (2018). Lipid biology in fungal stress and virulence: Entomopathogenic fungi. Fungal Biol..

[62] Strijbis K., Distel B. (2010). Intracellular acetyl unit transport in fungal carbon metabolism. Eukaryot. Cell.

